# Mining for novel candidate clock genes in the circadian regulatory network

**DOI:** 10.1186/s12918-015-0227-2

**Published:** 2015-11-14

**Authors:** Anuprabha Bhargava, Hanspeter Herzel, Bharath Ananthasubramaniam

**Affiliations:** Institute for Theoretical Biology, Charité Universitätsmedizin, Phillipstr. 13, Haus 4, Berlin, 10115 Germany; Institute for Theoretical Biology, Humboldt Universität zu Berlin, Invalidenstr. 43, Berlin, 10115 Germany

**Keywords:** Mammalian circadian clock, Clock genes, Meta-analysis, Phase regulation

## Abstract

**Background:**

Most physiological processes in mammals are temporally regulated by means of a master circadian clock in the brain and peripheral oscillators in most other tissues. A transcriptional-translation feedback network of clock genes produces near 24 h oscillations in clock gene and protein expression. Here, we aim to identify novel additions to the clock network using a meta-analysis of public chromatin immunoprecipitation sequencing (ChIP-seq), proteomics and protein-protein interaction data starting from a published list of 1000 genes with robust transcriptional rhythms and circadian phenotypes of knockdowns.

**Results:**

We identified 20 candidate genes including nine known clock genes that received significantly high scores and were also robust to the relative weights assigned to different data types. Our scoring was consistent with the original ranking of the 1000 genes, but also provided novel complementary insights. Candidate genes were enriched for genes expressed in a circadian manner in multiple tissues with regulation driven mainly by transcription factors BMAL1 and REV-ERB *α*,*β*. Moreover, peak transcription of candidate genes was remarkably consistent across tissues. While peaks of the 1000 genes were distributed uniformly throughout the day, candidate gene peaks were strongly concentrated around dusk. Finally, we showed that binding of specific transcription factors to a gene promoter was predictive of peak transcription at a certain time of day and discuss combinatorial phase regulation.

**Conclusions:**

Combining complementary publicly-available data targeting different levels of regulation within the circadian network, we filtered the original list and found 11 novel robust candidate clock genes. Using the criteria of circadian proteomic expression, circadian expression in multiple tissues and independent gene knockdown data, we propose six genes (*Por*, *Mtss1*, *Dgat2*, *Pim3*, *Ppp1r3b*, *Upp2*) involved in metabolism and cancer for further experimental investigation. The availability of public high-throughput databases makes such meta-analysis a promising approach to test consistency between sources and tap their entire potential.

**Electronic supplementary material:**

The online version of this article (doi:10.1186/s12918-015-0227-2) contains supplementary material, which is available to authorized users.

## Background

The daily and seasonal geophysical variations have driven the evolution of a circadian clock system in most organisms. These biological timekeepers permit organisms to maintain near 24 h rhythms in most physiological processes and anticipate periodic changes in their environments. In mammals, the circadian system consists of a master circadian timekeeper in the suprachiasmatic nucleus (SCN) in the hypothalamus [1] and several slave timekeepers distributed in multiple tissues throughout the body, such as the liver, lungs and kidney [2, 3]. Nevertheless, there is a common underlying mechanism producing circadian rhythms in these tissues based on transcriptional-translational feedback loops (TTFL) [4]. In the TTFL, the protein products of several genes inhibits their own transcription after associated delays due to transcription, translation and post-translational modification. After the identification of the SCN master clock [5], the first component of the TTFL was identified as the gene *Clock* [6].

Subsequently, other “core” members of the TTFL, such as the repressors *Period* (*Per1,Per2,Per3*) and *Cryptochrome* (*Cry1*,*Cry2*,*Cry3*), activators *Arntl* (*Bma1*) and *Npas2*, and nuclear receptors *Rev-erb* (*Nr1d1*, *Nr1d2*) and *Ror* (*Rora*, *Rorb*, *Rorc*) were established. There has been continued interest in finding new members of the TTFL not only for better understanding the mammalian circadian clock, but also because mutations in these core genes have been linked to several disorders [7]. While *Clock* was discovered by costly and laborious forward genetic screens by Joseph Takahashi and colleagues [6], current high-throughput data from genetics, transcriptomics and proteomics and availability of the entire genome combined with system biological approaches have tremendously accelerated our ability to find new putative members of the TTFL [8]. Recently, Anafi et al. used probabilistic machine learning to identify a putative clock member and subsequently experimentally verified it to discover the novel clock gene CHRONO [9]. Similar bioinformatic approaches were used to identify novel circadian genes from microarray data [10] and using co-expression data and text-mining [11], to find circadian genes disrupted in cancer cell lines [12] and to find health implications of disrupted clock genes [13].

In this work, we aim to filter the list of a 1000 putative clock genes from [9] to determine the strongest candidates for further experimental validation. We do this by including other sources of high-throughput data, such as chromatin immunoprecipitation sequencing (ChIP-seq), proteomic and protein-protein interaction (PPI) data, not included in the original machine-learning procedure of [9]. We combined metrics for different data sources using a simple scoring scheme and shortlisted P450 cytochrome oxidoreductase (*Por*), metastasis suppressor 1 (*Mtss1*), proviral integration site 3 (*Pim3*), Diacylglycerol O-acyltransferase 2 (*Dgat2*), protein phosphatase 1 regulatory sub-unit 3b (*Ppp1r3b*) and uridine phosphorylase 2 (*Upp2*).

## Method

We started our meta-analysis from the list of 1000 putative clock genes identified by Anafi et al. (Table S2 in [9]), henceforth referred to as the ‘master list’. Anafi and colleagues compiled this master list by combining Bayesian scores representing five features necessary in a clock gene: (i) oscillating transcripts in liver, pituitary and NIH3T3 cells; (ii) a circadian phenotype in response to RNA interference (RNAi) of the gene; (iii) significant number of functional genetic interactions with an exemplar list of known “core” clock genes based on radiation hybrid mapping; (iv) ubiquity of expression of the gene across multiple tissues based on expressed sequence tags (EST); (v) phylogenic conservation across fruit flies (*Drosophila melanogaster*) and mammals. We exploit the wealth of available high-throughput genomic, transcriptomic and proteomic data (Table 1) on the circadian system not used by Anafi et al. to further screen the master list for putative clock genes that are most worthy of experimental validation. We restricted our attention to data on mice (*Mus musculus*), since circadian data sets are overwhelmingly based on the mouse model and therefore several time-resolved high quality circadian data sets are available (as opposed to other mammals). However, we included data from all mammals in the non-circadian PPI data sets. Our data set selection criteria was to include all ChIP-seq data on circadian TFs, circadian proteomics data and PPI data on mice that was available when this meta-analysis was conducted.

**Table 1:** Data sources used in this study

Type	Source	Characterized	Total	Circadian	Hits
	Koike et al. [14]	BMAL1	3495	3004	359^ns^
		PER1	2984	138	15^ns^
		PER2	4255	3495	384^ns^
		CRY1	6768	2923	356^***^
		CRY2	5230	2717	318^**^
		CLOCK	2831	1204	170^***^
ChIP-seq		NPAS	1597	808	121^ns^
	Rey et al. [15]	BMAL1	1273	439	228^*^
	Cho et al. [16]	REV-ERB *α*	3849	-	412
		REV-ERB *β*	3849	-	412
	Bugge et al. [21]	REV-ERB *α*	6256	-	636
	Feng et al. [22]	REV-ERB *β*	6444	-	635
	Fang et al. [23]	ROR *α*	8457	-	529
		E4BP4 (NFIL3)	6147	-	437
Proteomics	Robles et al. [25]		2877	185	34^**^
	Mauvoisin et al. [26]		5610	193	35^***^
	Chiang et al. [27]		1881	47	6^ns^
Protein-protein interaction	Wallach et al. [28]		123	-	25
	PINA mouse database [31, 32]		467	-	33

The source of the published data, the characterized protein or transcription factor, the total number of binding sites for the ChIP-seq data and the number of genes corresponding to the quantified proteins in the case of proteomics or protein-protein interaction data are provided. When data could be filtered to include only circadian components, the number of genes with circadian ChIP-seq or proteomic evidence is also listed. Finally, the total number of hits from each data set among the 1000 gene long master list is given. The statistically overrepresented circadian hits are marked by significance (see “Method” section) ^∗^:*p*<0.05,^∗∗^:*p*<0.01,^∗∗∗^:*p*<0.001,^*ns*^:*not significant*

### ChIP-seq data

The rhythm generation in the circadian clock is a result of a TTFL, as described earlier. Within the TTFL, clock genes acting as transcription factors mutually regulate each other. Thus, it is likely that putative clock genes are transcriptionally regulated by known clock transcription factors (TFs), such as BMAL1, CLOCK, NPAS2, E4BP4, ROR *α*, REV-ERB *α* and REV-ERB *β*. Therefore, we searched for ChIP-seq binding sites for each clock TF in the vicinity of the genes in the master list. Moreover, when time-resolved binding of the TF factor was measured in a study, we restricted our attention further to only those ChIP-seq binding sites that show circadian (cycling) binding of the TF.

Koike et al. [14] provide time-resolved genome-wide ChIP-seq binding sampled every four hours over one day in mouse liver for the TFs BMAL1, CLOCK, NPAS, CRY1, CRY2, PER1 and PER2 (the list of ChIP-seq peaks and associated genes for each TF were provided in Table S2 of [14]). It is worth noting that PERs and CRYs do not possess DNA binding domains, but we still retain the data as we believe that they might represent regulation by a complex composed of these important circadian proteins. We used the false discovery rates (FDR) for circadian binding from the original study to filter genes with circadian TF binding using a cutoff of 0.05.

Rey et al. [15] similarly performed a detailed study of the genome-wide binding of activator BMAL1 in the mouse liver sampled every four hours over one day (the list of ChIP-seq peaks and associated genes for each TF were provided in Text S2 of [15]). As before, we restricted our choice to gene with circadian BMAL1 binding with a FDR<0.05, based on a Fisher test for periodicity as suggested by the authors of the study.

Cho et al. [16] compared the cistromes of both isoforms of the nuclear receptor repressor REV-ERB (*α* and *β*) at a single time point (Zeitgeber time (ZT) 8) and against the cistrome of BMAL1. The raw ChIP-seq (data accessible at NCBI GEO database [17–19], accession GSE34019) was processed using the TFTargetCaller [settings: Ouyang, TFAS and closestGene methods] package in R [20] to produce an annotated list of ChIP-seq peaks. Although they discovered significant overlap between the binding sites of both isoforms, we retained the genes proximal to binding sites of each isoform, separately.

Bugge et al. [21] and Feng et al. [22], in related studies, investigated the role of nuclear-receptor repressors REV-ERB *α* and REV-ERB *β* in metabolism in the liver. They measured binding using ChIP-seq of both TFs at two time points, 12 hours apart. As before, we processed the raw ChIP-seq data (NCBI GEO database [17], accession GSE36375 and GSE26345, respectively) using the TFTargetCaller [settings: Ouyang, TFAS and closestGene methods] package in R [20] to produce an annotated list of ChIP-seq peaks. Since circadian binding cannot be reliably determined from two time points, we pooled binding sites from both time points for both TFs.

Fang et al. [23], recently, measured binding of the D-box repressor E4BP4 (NFIL3) and nuclear receptor activator (ROR *α*) in the mouse liver at ZT22 as part of a study on identifying functional circadian ChIP-seq binding sites. We processed their raw data (data accessible at NCBI GEO database [17], accession GSE59486) using HOMER (v4.7.2) [24] for peak-calling and assigning peaks to the nearest gene.

### Proteomics data

A common feature of clock genes is that they are cycling both at the transcript as well as the protein level. The circadian nature of the former is already included in compiling the master list. Subsequently, three studies of the circadian proteome were published and we use them as another source of data for screening.

Robles et al. [25] quantified the circadian proteome in the liver sampled at 16 time-points over two days under light-dark conditions. They used a stable isotope labeling by amino acids in cell culture (SILAC) combined with mass spectrometry (MS) to find oscillating proteins. We included all genes whose proteins were determined to have significant oscillations with a 0.32 FDR cut-off in the original study (the statistically significant circadian proteins are listed in Table S2 in [25]).

Mauvoisin et al. [26] performed a similar study of the liver proteome under light-dark conditions also using the SILAC-MS approach. With 16 samples over two days, we selected those gene whose proteins were deemed circadian with an FDR of 0.25 according to the analysis in the study (Dataset S1 in [26] contains the list of characterized proteins with FDR values from the rhythmicity analysis).

Chiang et al. [27], on the other hand, quantified the mouse SCN proteome sampled at six time points over one day using SILAC combined a slightly different MS approach. The genes with time-of-day (circadian) variation in protein concentration were selected using a 0.05 FDR cut-off (the list of all characterized proteins with a *p*-value for their rhythmicity can be found in Table S2 in [27]).

It is interesting to note that none of the proteomics studies identified as circadian the core circadian proteins, such as the PERs and CRYs, since their levels were too low to be detected using current techniques.

### Protein-protein interaction data

For a gene to be an integral part of the TTFL, it might interact at the protein level with known clock members. Therefore, we complemented the gene interaction study used by Anafi et al. with publicly available protein-protein interaction data. In particular, we searched for protein interactions with a list of 46 core circadian genes gathered by Wallach et al. [28]. Note that this list of core circadian genes is longer than the exemplar list of 17 used by Anafi et al. [9].

Wallach et al. [28] compiled the list of all proteins interacting with the 46 core circadian genes from the Unified Human Interactome (UniHi) database [29] that was mined from 14 different sources using four different approaches. We looked for evidence of interactions between genes in the master list and these 46 core clock genes (the list of interactions of the core clock genes was provided in Table S1 in [28]).

From the Protein Interaction Network Analysis (PINA) database [30–32], we were able to obtain complementary data on interactions identified in *M. musculus* alone that is similarly drawn from multiple public resources. As before, genes in the master list with at least one interaction with the 46 core clock genes were sought in the PINA database.

### Combining the sources and scoring

We drew our data from multiple sources that are generated using different techniques, as described above, which makes integrating them a non-trivial task. It is particularly difficult to assign confidences to these different measurements. We therefore resorted to a very simple scoring scheme where the presence of a gene in a particular data set (termed a ‘hit’) gets a score of one and its absence, a score of zero, otherwise. Thus, we give equal weight to a data set, whether ChIP-seq, proteomic or PPI. The only exception to this weighting rule is the data set from Rey et al. [15], which is given a weight of three, since it is a particularly detailed and thorough study of a central transcription factor (BMAL1).

Whenever time-resolved data were available, we restricted our attention to binding, concentrations or interactions that were identified to be circadian at the appropriate statistical level (see details above). We mapped all protein data back to coding gene and assigned ChIP-seq peaks to the nearest gene, and used the ENTREZ gene ID as the common unified index for each gene (using Bioconductor annotation package org.Mm.eg.db (v3.1.2)). The final score for each gene in the master list was computed as the sum of gene score within each constituent data set and reflects simply the number of studies that provide evidence supporting the circadian nature of that gene. A gene under this scheme can receive a maximum score of 21.

We were able to attach a *p*-value to the total score for each gene using the following randomized shuffling procedure. We assigned the total number of hits obtained from each data set randomly to genes in the master list. Iterating this procedure, we produced an empirical distribution of scores and the probability of achieving a more extreme total score (the *p*-value) can be thus computed.

### Selecting genes robust to the choice of weighting scheme

We used a simple binary scoring scheme for each gene within a data set that resulted in a much heigher weight being assigned to the ChIP-seq data compared to the other two. We therefore tested if genes were particulary sensitive to the relative choice of weights for the different types of data sets. We therefore introduced pre-factors for each class of data: *α* for ChIP-seq, *β* for proteomic, and *γ* for protein-protein interaction (PPI), respectively. Then, the total score for a gene *g* for this choice of pre-factors is

$$\begin{aligned} S(g)&= \!\alpha~\![\textrm{\!ChIP-seq data score}] \!+ \beta~[\!\textrm{Proteomics data score}]\\ &\quad + \gamma~[\textrm{PPI data score}] \\ &=\alpha \!\!\sum_{\substack{\textrm{ChIP-seq}\\ \text{data}~i}} I_{i}(g) + ~\beta\!\! \sum_{\substack{\text{Proteomics} \\ \text{data}~ j}} I_{j}(g) + ~\gamma \sum_{\textrm{PPI data }k} I_{k}(g), \end{aligned} $$

where *I*_*l*_(*g*) is one, if the gene *g* is present in a data set *l*, zero if it is not. We further fix *α*+*β*+*γ*=1.

**Fig. 1:**
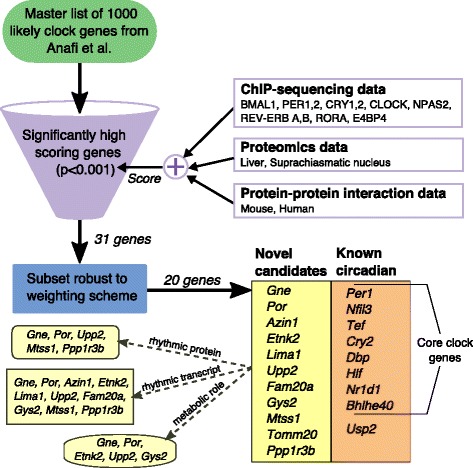
Data mining approach to find novel clock candidate genes. The schematic outlines the approach along with the types of data sources used to filter the master list of 1000 genes from Anafi et al. [9] to the list of 11 novel candidate clock genes. Some key properties of the novel candidates genes are also indicated. The entire list of robust and non-robust candidate genes are given in Table S1 in Additional file 2

Our original scoring described above and in Additional file 1 is equivalent to a choice of equal pre-factors for all three classes, i.e., $\alpha = \beta = \gamma = \frac {1}{3}$. We then altered each pre-factor randomly while restricting changes to at most 50 % above or below the original equal pre-factors. In other words, we generate random (*α*,*β*,*γ*), such that 0.165<*α*,*β*,*γ*<0.5. Care was taken while generating these random pre-factors to ensure that the pre-factors were truly uniformly distributed over the simplex. Finally, for each random choice of pre-factors, we computed a background distribution of scores *S* using the randomized shuffling procedure while keeping the (*α*,*β*,*γ*) fixed. Significant candidate genes for that particular choice of pre-factors were those genes with a *p*-value <0.001. Finally, we retained only genes that were found to be significant in each of 100 random choices of (*α*,*β*,*γ*). This list of genes was thus robust to variations in the choice of pre-factors and are listed in Fig. 1.

### Model of phase regulation of genes by combinations of transcription factors

We processed the mRNA transcript time series data from [33] (data accessible at NCBI GEO database [17], accession GSE54650) using JTKcycle [34] to obtain phases of master list genes. We then grouped the ChIP-seq data sets (Figure S1 in Additional file 2) into E-box activators (CLOCK, BMAL1 and NPAS2), D-box repressor (E4BP4), and RRE regulators (REV-ERB *α*,*β*, ROR *α*). We used the ChIP-seq components in the table of scores (Additional file 1) to construct scores for each TF group regulating a gene by summing all ChIP-seq scores for that TF group in the table of scores and normalizing each score by the maximum score attainable within that TF group. Each gene was thus associated with three scores, *S*_E_, *S*_D_ and *S*_RRE_, for the E-box, D-box and RRE TF groups, respectively, that all take values between zero and one.

We fit a type of generalized linear model (GLM) for the phase of gene transcripts with the three scores as predictors. Since transcript phase is a circular dependent variable (i.e., phase CT24 is equal to CT0), we needed a link function to relate the linear predictor based on the three scores to the transcript phase. The circular variable is assumed to have a von Mises distribution whose mean is linked to the linear regressors via an ‘$\arctan $’ link function as suggested in [35]:

$$ E(\phi_{\text{gene}}) = \mu + 2\arctan\left\{ c_{\mathrm{E}}~S_{\mathrm{E}} + c_{\mathrm{D}}~S_{\mathrm{D}} + c_{\textrm{RRE}}~S_{\textrm{RRE}} \right\},   $$

where *ϕ*_gene_ is the wrapped gene phase in CT and the model parameters include the unregulated mean phase *μ* of the genes and the contributions of the three scores to the linear regressor (*c*_E_,*c*_D_,*c*_RRE_). The concentration parameter of the von Mises distribution *κ* is assumed to be constant and independent of the TF scores and is also estimated (this parameter behaves like the inverse of the standard deviation). The GLM was fit using iteratively-weighted least squares implemented in the *circular* package in R.

## Results

### candidate genes identified by the meta-analysis

The meta-analysis approach outlined in Fig. 1 yielded a table of scores for the 1000 genes in the master list (Additional file 1), which is one of the primary results of this work. The ChIP-seq data sets contributed the largest number of hits to the master list and hence, was the largest contributor to the total score (Table 1). The proteomic and PPI data sets had limited effect on the total score. Based on the ChIP-seq data sets, genes in the master list were mostly bound by transcription factors (TFs) via RRE and to a lesser extent at E-boxes and D-boxes (Additional file 3: Figure S1). It must be noted, that the E-box binding TFs satisfied the stringent criteria of binding chromatin in a circadian manner, while the rest were based on experiments performed at one or two circadian phases.

We tested whether the presence of a gene in any of the data sets assembled in this study is predictive of high ranks in the original evidence factor-based ranking of Anafi et al. [9]. Interestingly, presence of circadian BMAL1 binding [14] (*p*<0.05), or circadian proteome according to [26] (*p*<0.01), or identified PPIs with any clock protein in mouse or humans (*p*<0.05) were all significantly predictive of high ranks. Overall, we found a significant but weak positive association between the original ranking and a ranking based on our score (correlation = 0.12, *p*<0.001). Thus, the data sets incorporated in this work are broadly consistent with the master list from [9].

Thirty-one genes in the master list (listed in Table S1 in Additional file 2) obtained statistically significant total scores based on the randomized shuffling procedure, where a total score of 13 was significant at the 0.001 level (Additional file 3: Figure S2). We used this rather stringent threshold to compensate for the inherent correlation in the scores between data sets (for e.g., we have multiple REV-ERB and BMAL1 ChIP-seq data) that is unaccounted for in the shuffling procedure.

Our simple scoring of genes results in ChIP-seq data sets contributing the most to the total score of each gene, as described above. We therefore compensated for this effect by pruning out genes from the list of 31 statistically significant scoring genes that were not robust to the choice of relative weights for the ChIP-seq, proteomic and PPI data sets (see “Method” section). After pruning, we were left with 20 robust candidate clock-associated genes (Fig. 1) that included nine known clock-associated genes. In fact, all the known clock-associated genes in the list of significant scorers (Table S1 in Additional file 2) were robust to the weighting scheme. Further, our candidate list contained 8 out of 18 ‘core’ clock genes defined by Ueda and colleagues [36, 37] (see Table S2 in Additional file 2).

**Fig. 2:**
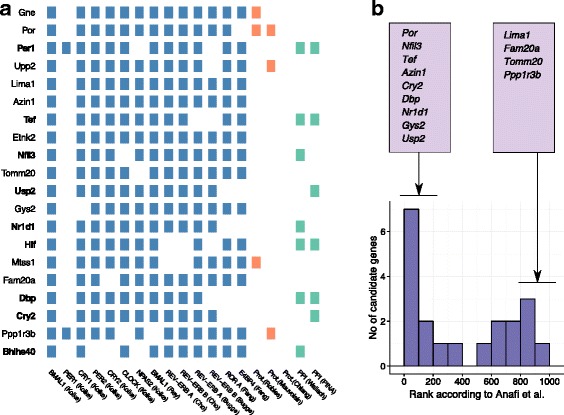
Scores of the significant and robust candidate genes and their relationship to the original ranking in Anafi et al. **a** The table of individual hits within the ChIP-sequencing, proteomics and protein-protein interaction data sets (in blue, orange and green, respectively) for the 20 significant candidate genes (at the 0.001 level) based on our meta-analysis that are also robust to the choice of weighting schemes for the different data sets. The nine known clock-associated genes are marked in bold on the y-axis. **b** The distribution of the evidence-based ranks from Anafi et al. [9] for the 20 significant and robust candidate genes identified in **a**. The candidate genes that obtained evidence-based ranks in the top and bottom 200 are also listed

Among the 11 novel robust candidate genes, all except *Tomm20* had a rhythmic transcript in at least one tissue [33] and five genes (*Por*, *Gne*, *Upp2*, *Mtss1*, *Ppp1r3b*) had a significantly rhythmic protein product in at least one of the three proteomic studies (Fig. 2a). In line with the overall trends identified earlier, most novel genes were predominated by ChIP-seq binding of clock TFs with little evidence in the proteomic or PPI studies. Moreover, 9 of the 20 robust candidate genes featured in the top 200 ‘circadian’ genes in [9] including six known clock genes (Fig. 2b). The novel robust candidates in the top 200 were *Por*, *Azin1*, and *Gys2*. Curiously, we observed a bimodal distribution of evidence-based ranks among these 20 robust candidates (Fig. 2b), with genes falling in the top or bottom of the Anafi et al. list. *Lima1*, *Fam20a* and *Ppp1r3b* constitute robust candidates that were indeed ranked in the bottom 200, where our meta-analysis adds novelty to the candidate list.

Motivated by the close association between the circadian clock and metabolism, we checked if candidate gene products catalyzed metabolic reactions using the KEGG database [38, 39]. *Por*, *Gne*, *Upp2*, *Gys2* and *Etnk2* were robust candidate genes with enzymatic functions, while *Dgat2*, *Pik3r1* and *Chka* were significantly high-scoring, but non-robust, genes with a metabolic role. However, only *Upp2* and *Gys2* were directly involved in catalyzing reactions with at least one substrate with circadian variation in abundance (Uracil and UDP-galactose, respectively) in the mouse liver [40]. However, no substrate of candidate genes were circadian in the human metabolome in the saliva [41].

### Candidate gene transcripts in different tissues

We next compared our meta-analysis to the recently published high-resolution micro-array and RNA-sequencing study of the circadian transcriptome in 14 tissues [33]. Recall that the original master list was compiled using an earlier microarray circadian transcriptome study in just three tissues [42] and with the intention of finding genes with ubiquitously circadian transcripts. Therefore, it was surprising to find that only 728 out of the 1000 master list genes had a circadian transcript (FDR_JTK_<0.05) in at least one tissue in [33].

Nevertheless, the total score was reasonably correlated (Pearson’s correlation =0.44, *p*<0.0001) with the number of tissues in which the gene transcript was significantly circadian (FDR_JTK_<0.05), even with the simple scoring methodology used here. When we considered individual components of the score, we found that E-box binding, circadian protein expression and PPI with clock genes to be the most significant predictors of genes transcribed in a circadian manner in multiple tissues (*p*<0.001), though RRE binding also played a, albeit less significant, role (*p*<0.01). Moreover, BMAL1 and REV-ERB *α*,*β* appeared to be the main E-box and RRE transcription factors, respectively, responsible for driving oscillations in many tissues.

**Fig. 3:**
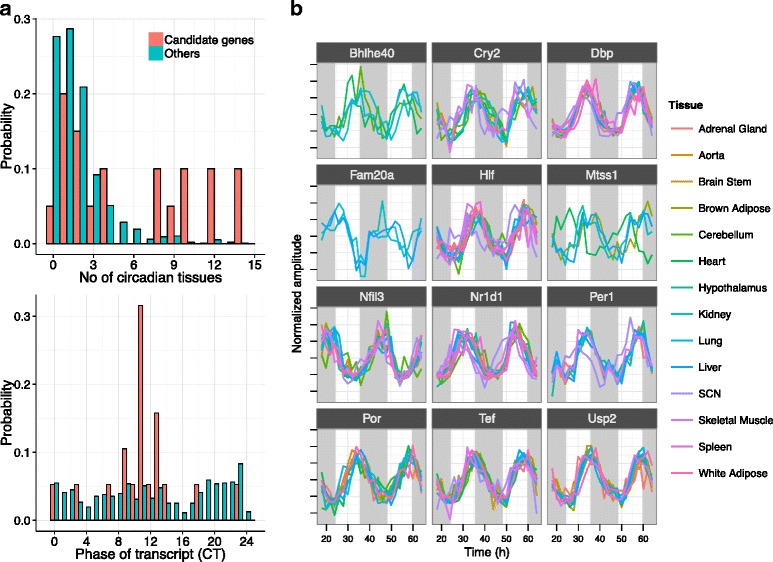
Transcript expression of candidate genes. **a** The distributions of the number of different tissues and expression phase in those tissues compared across candidate genes (red) and the rest of the master list (green) based on [33]. **b** The circadian expression profiles in fourteen different tissues (data from [33]) of genes in the candidate list that were expressed in a circadian manner in at least four tissues

The 20 robust candidate genes were significantly over-representative of genes that were circadian in many tissues (two sample two-sided Kolmogorov-Smirnov test, *p*<0.005) (Fig. 3a). More than 50 % of the candidate genes were expressed in a circadian manner in at least four tissues, while this reduces to 13 % among the low-scoring non-robust non-significant genes (Table S3 in Additional file 2). In addition, when robust candidate genes were sorted in descending order of the number of tissues in which they showed circadian transcripts, eight of the nine known clock-associated genes were at the top of this list. Interestingly, significantly high-scoring non-robust genes were also generally expressed in many tissues and exhibited similar properties to the robust candidates (Table S3 in Additional file 2).

When we consider the regulated phases of only significant circadian transcripts (we pooled data from all 14 tissues), we noticed that genes in the master list were expressed uniformly throughout the day with a slight bias towards CT23-CT24/0 (Fig. 3a). However, transcript phases (time of peak expression) of robust candidate genes were strongly concentrated around CT9-13 (Fig. 3a). Remarkably, the circadian transcripts of candidate genes in different tissues shared the same phase of peak expression (see Table S3 in Additional file 2) with very few exceptions. In fact, candidate genes (both robust and non-robust) that were circadian in at least five tissues showed a very high degree of expression phase coherence (Fig. 3b). The exceptions were mostly genes expressed in four or fewer tissues, such as *Chka* and *Mtss1*. For example, *Mtss1* and *Chka* showed distinctly different phase of expression in the heart as compared to the other three tissues in which they were expressed in a circadian manner (Fig. 3b).

### Regulation of master list genes by circadian transcription factors

In the mammalian transcriptome, few circadian TFs appear to regulate many circadian transcripts with a wide variety of expression phases throughout the circadian cycle (e.g., Fig. 3a). One hypothesis of circadian phase regulation is that different circadian genes are regulated by different subsets (combinatorial) of TFs. This results in different phases of expression of the regulated genes, since each circadian TF has its peak expression at a different time of day (phases). We examined this hypothesis of whether binding of certain TFs was indicative of phase-specific transcript regulation using the ChIP-seq data on different circadian TFs and the transcript phase of master list genes. In particular, we assumed in this analysis that ChIP-seq binding of TFs in the mouse liver is highly representative of binding of those TFs in other tissues. This allowed us to pool circadian phases of significant transcripts from all 14 tissues [33] in order to get sufficient sample sizes in each combination of TFs.

We grouped the ChIP data sets (Figure S1 in Additional file 2) into E-box activators (CLOCK, BMAL1 and NPAS2), D-box repressor (E4BP4), and RRE regulators (REV-ERB *α*,*β*, ROR *α*). We were able to combine RRE activator and repressors, since the RRE activators and repressors were expressed 12 h apart (i.e., out-of-phase) (see Table S4 in Additional file 2). In other words, the peak of the RRE activator and nadir of the RRE repressor occur approximately simultaneously and vice-versa, thus, making their effects on gene transcription similar. Based on this classification, we expect activation via each regulator group to peak when the corresponding TF peaks (see Table S4 in Additional file 2).

**Fig. 4:**
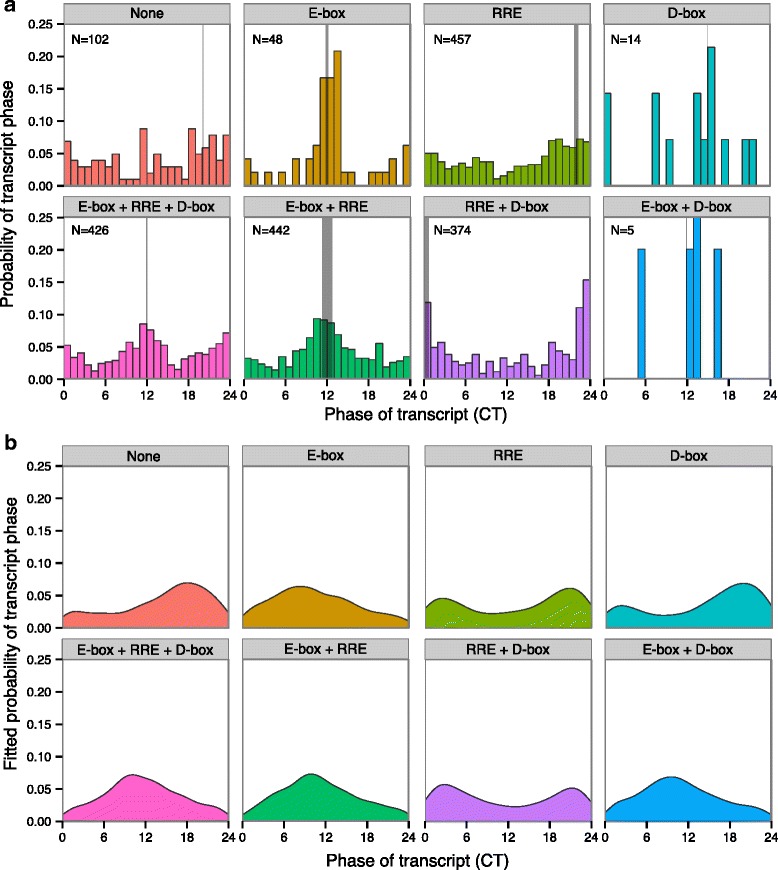
Phase regulation of circadian transcripts. **a** The distribution of the expression phase of genes that are significantly circadian in different tissues (FDR_JTK_<0.05) [33] classified according to the combinations of transcription factors (TFs) binding their promoter based on ChIP-seq data. E-box: BMAL1 or NPAS2 or CLOCK, D-box: E4BP4, RRE: REV-ERB *α* or REV-ERB *β* or ROR *α*. The grey vertical lines represent the median phase of expression within each group of transcripts and the line width increases with increasing significance of the mean-direction (Rayleigh test). The number of transcripts in each group is also provided. **b** The estimated distribution of phases for each combination of TFs from the generalized linear model fit of the transcript phases to the normalized scores for each TF group within the ChIP-seq data in our meta-analysis. Under the model, the phase of expression within each group is assumed to follow a von Mises distribution, whose mean is linearly dependent on the normalized scores (see “Method” section)

We first qualitatively compared the presence or absence of these regulatory features of genes against the phase distribution of the transcripts (Fig. 4a). Transcripts regulated by only E-box elements were likely to peak around dusk (CT10-14), while only RRE regulated transcripts peaked commonly around dawn (CT22-24/0). For reference, *Rev-erbβ* is a typical E-box gene that peaks at C10 and *Bmal1* is a typical RRE regulated gene that peaks at CT0. Genes in this list regulated by both elements showed a spread of transcripts phases throughout the day with a bias towards dusk-phased E-box genes. Few genes in the master list were regulated either by D-boxes alone or by a combination of E- and D-boxes and hence, no conclusions could be drawn on them. The confluence of repression by a dusk-phased D-box regulator and RRE regulators produced an even stronger peak of expression at CT22-0. Finally, regulation by all three groups of TFs produced two equally strong peaks of expression at dawn and dusk.

We computed normalized scores for the regulation of each gene by the different TF groups based on the ChIP-seq data (number of hits) and then a regression between the expression phase of the gene (in CT) against a linear combination of the scores (see “Method” section). We assumed that genes with higher scores, i.e., more consensus within the ChIP-seq data sets for a TF, also have higher affinity for the particular TF. The fitted model of phase regulation was as follows:

(1) $$ \begin{aligned} \textrm{Mean gene phase (in CT)} &= 17.97 + 2\arctan\left\{ -2.25~S_{\mathrm{E}}\right. \\ &\quad\left.+ 0.67~S_{\mathrm{D}} + 0.41~S_{\textrm{RRE}} \right\},  \end{aligned}  $$

where *S*_E_,*S*_D_,*S*_RRE_ are the ChIP-seq scores for E-box, D-box and RRE regulators respectively. The model parameter estimates were significant at the 0.001 level and the concentration parameter of the phase distribution was estimated to be 0.58 (this parameter behaves like the inverse of the standard deviation).

The predicted phase distribution for genes regulated by different combination of TFs from the fitted model in (1) is shown in Fig. 4b. The model fits are consistent with the qualitative observations in Fig. 4a. The model Eq. 1 reveals that the mean expression of genes unregulated by these TFs in the master list is CT18. Genes with higher E-box scores progressively decrease the gene phase from CT18, i.e., move them towards dusk. On the other hand, D-box and RRE regulation increase the gene phase from CT18, i.e., move them towards dawn. However, E-box scores have a larger effect on phase than D-box and RRE scores. Finally, the model can be used to predict the phase of expression for other genes, if ChIP-seq data are available to estimate their scores.

## Discussion

We presented in this paper a meta-analysis (Fig. 1), where we integrated circadian ChIP-seq, proteomics and protein-protein interaction (PPI) data with the recently published machine learning-based selection of putative circadian genes from transcriptomic data, small interference RNA (siRNA) screens and genomic conservation data [9]. Our goal was to screen for novel candidate genes bioinformatically using publicly available data sources. This analysis produced a table of scores (Additional file 1) for the 1000 potential circadian genes from [9]. Interestingly, we found no strong correlation between our ranking based on gene scores and the ranking of Anafi et al., suggesting that two sets of sources provide complementary insights into the circadian nature of the genes. The Anafi et al. ranking reflects mainly transcriptional rhythmicity and as such the ChIP-seq data would be expected to be consistent with it, since they relate to the same process of transcription. However, binding to the chromatin is not always functional and unless the activity of the TF is also circadian, binding of circadian TFs does not necessitate circadian transcription. This nevertheless represents an inherent limitation of ChIP-seq data and is a caveat to our entire study. On the other hand, proteomics and PPI are truly independent data sources, since they represent altogether different cellular processes from transcription. We therefore expect that our three data sources do provide new information as compared to [9] to further filter these genes.

The ChIP-seq data consisted of the largest number of different sources and predictably had the strongest influence on the scores. The three categories of TFs, E-box binding, RRE binding and D-box binding were all individually influential and gave hits in the master list (Table 1). Although there were few genes in the master list with a circadian protein expression, they were significantly over-represented in the master list. This can be attributed to the few circadian proteins identified overall in those studies [25, 26], where low concentrations make quantification of most known core circadian genes difficult. The PPI with known core clock genes also provided sparse hits within the 1000 master list genes. Nevertheless, hits from PPI, BMAL1 and REV-ERB *α*,*β* ChIP-seq and circadian proteomics data sets were predictive of both original evidence-based ranking and rhythmic transcriptome in the mouse liver, pituitary and NIH3T3 cells as quantified by [9]. Thus, despite the low correlation between scoring methodologies alluded to earlier, the newly integrated data appear to be consistent with the original data sources.

We verified our approach against the recently published RNA-sequencing study on the circadian transcriptome in multiple tissues [33], which was not used in our study. Surprisingly, only about 70 % of genes in the master list were circadian in at least one of fourteen tissues. Nevertheless, we found that high gene scores in our meta-analysis was indicative of robust circadian transcripts in multiple tissues. This supports the ability of our analysis methodology to find potential clock-associated genes, since we expect candidate clock genes to be ubiquitously circadian. Consistent with conclusions based on data of [42], we found BMAL1 and REV-ERB *α*,*β* were predictive of circadian rhythms in multiple tissues along with interaction with known circadian proteins and oscillating protein expression. This suggests that E-boxes and RRE repressors might be major drivers of the circadian transcriptome in multiple tissues.

We therefore compared the phases of the robust circadian transcriptome (FDR <0.05) in multiple tissues in [33] against the analyzed ChIP-seq data for E-box activators, D-box repressor and RRE regulators in order to check for characteristic patterns of phase regulation by these TFs. One remarkable feature was that the transcript phase for at least the master list genes were quite consistent across 14 tissues. Consistent with expectations, we found that genes with only RRE binding were likely to peak around dawn (CT0), whereas E-box only bound genes were likely to peak at around dusk (CT12). Those genes with binding at both E-boxes and RREs showed a mixture of phases between dawn and dusk. Moreover, we observed that the D-box repressor regulated most master list genes only in conjunction with RREs, where they enhanced dawn-phase expression, since the effect of both regulators were in-phase. Indeed, Fang et al. [23] showed that this D-box repressor (E4BP4) works downstream of Rev-erb *α*,*β*. The presence of all three regulator types produces both dawn and dusk-phase expression.

The amount of consensus between the different ChIP-seq sources in our meta-analysis was used to measure the affinity of TFs to particular genes as a score. The E-boxes, D-boxes and RREs scores for each gene were then used as predictors of the expression phase of the gene via a model. This model provided a statistically significant fit to the data and described how the presence of the different TFs quantitatively tuned the expression phase of a gene (Fig. 4). It must be borne in mind that for this analysis of phase regulation, we pooled all the transcript phases and assumed that ChIP-seq binding in the liver implies similar binding in all tissues. Moreover, since the master list was itself obtained from a machine-learning-based search for novel clock genes [9], this list might not be representative of the entire circadian transcriptome.

We found 20 candidate genes with highly significant scores in our meta-analysis that were also robust to the choice of relative weights for the ChIP-seq, proteomics and PPI data. Nine known clock-associated genes were included in these 20 genes, serving as a sanity-check for our approach. Although there were abundant ChIP-seq hits among these robust candidate genes, the few PPI hits were present only for known clock genes and circadian protein expression was restricted to *Por*, *Gne*, *Mtss1*,*Upp2* and *Ppp1r3b*. The intersection of the top 200 genes in [9] and these candidate genes yielded four novel genes: *Por*, *Azin1*, and *Gys2*. Most known clock-associated robust candidates received high ranks in [9], while others that received quite low ranks were the truly novel candidates identified in this study, such as *Mtss1* and *Ppp1r3b* (Fig. 2b). Several novel robust candidate genes had protein products that had an enzymatic role in metabolism. This is not unexpected as most of the data used in our meta-analysis were gathered from the liver, which is known to have a very important role in metabolism. However, only *Upp2* and *Gys2* catalyzed reactions with a substrate with circadian variation in abundance in the mouse liver [40] and none of the substrates were circadian in humans [41].

These 20 robust candidate genes were also statistically expressed in more number of tissues than other master list genes based on circadian transcriptomics data [33]. Since known clock genes are generally expressed in many tissues, this is indeed a desirable feature for candidate clock-associated genes. In addition to the remarkable consistency of expression phases of gene transcripts in tissues in which they are significantly circadian, transcripts of candidate genes were enriched for peaks at dusk (CT11) suggestive of E-box regulation. The similarity in the phase of candidate genes across tissues sets them apart from circadian output genes that are suggested to be regulated in a tissue-specific manner [43].

We next discuss specific candidate genes that might be the most rewarding to be studied experimentally based on a combination of factors including circadian protein expression, number of tissues in which their transcript is circadian (Table S3 in Additional file 2) and their robustness to the choice of weights for the different data classes (Table S1 in Additional file 2 and Fig. 1).

NADPH-Cytochrome P450 oxidoreductase (*Por*) had a rhythmic transcript in nine tissues (liver, adrenal gland, brown and white adipose tissue, lung, heart, kidney, aorta and cerebellum) with an average phase of CT17 and was the only gene in our candidate gene list with a circadian protein concentration in both liver proteomic data sets. POR is a flavoprotein responsible for electron transfer from NADPH to all P450 enzymes in microsomes. While *Por* knockout in mice is embryonic lethal [44], conditional knockout of *Por* in the liver affects lipid metabolism and homeostatis and results in hepatic lipidosis [45]. Recently, Johnson et al. [46] showed that ablation of the liver circadian clock affects the levels of *Por* expression, protein and activity, which in turn likely plays in role in regulating hepatotoxicity caused by acetaminophen. Based on the analysis of the ChIP-seq data, the *Por* promoter is bound by three types of clock TFs, E-box regulators (CLOCK/NPAS2 and BMAL1), RRE regulators (REV-ERBs and ROR *α*) and D-box repressor (E4BP4).

Metastasis supressor 1 (*Mtss1*) was expressed in a circadian manner in four tissues (liver, heart, kidney and brown adipose tissue) with an average phase of CT15 and cycling protein levels in one liver study [25]. *Mtss1* was identified as a potential tumor suppressor as it was not expressed in human bladder cancer cell lines [47] and involved in signaling in other carcinomas. It also plays a role in cytoskeleton dynamics by interacting with actin filaments [48]. Since it is strongly expressed in the liver, *Mtss1* was identified as circadian gene with different phase of expression in rats and mice based on early microarray transcriptomic studies [10]. Moreover, *Mtss1* was only candidate gene that displayed significantly different phase of expression between tissues. While the *Mtss1* gene promoter certainly binds E-box regulators (CLOCK/NPAS2 and BMAL1), RRE activator (ROR *α*) and D-box repressor (E4BP4), the evidence of regulation by the RRE repressors (REV-ERB *α*,*β*) was mixed between two studies [16, 21].

Another oncogene, proviral integration site 3 *Pim3* was expressed with consistent phase (average of CT15) across six tissues without being detected in either proteomic study. *Pim3* encodes a kinase that is upregulated in many cancer cell lines [49] and downregulation of PIM3 retarted cell proliferation in human hepatoma cell lines [50]. Further, *Pim3* appears to play a role in glucose homeostatis by downregulating insulin-secretion in response to glucose, thus making *Pim3*^−/−^ mice tolerant to glucose *in vivo*. In the circadian context, *Pim3* was identified as a light-induced immediate-early gene in the SCN [51] making it important also in the function of master circadian clock consistent with the bioinformatic prediction that *Pim3* has a conserved CRE element its promoter [10]. The promoter of *Pim3* binds D-box repressor (E4BP4) and the RRE-regulators (REV-ERBs and ROR *α*), but there is inconsistency in the binding of E-box TF (BMAL1) between the two studies.

Diacylglycerol O-acyltransferase 2 *Dgat2* did not exhibit any cycling in protein levels, but showed transcript oscillations with an average phase of CT17 in six tissues. *Dgat2* is responsible for the synthesis of triglycerides from diacylglycerol. Moreover, *Dgat2* is directly regulated by the cardiomyocyte circadian clock and participates in the response of the heart to fatty acids [52]. The E-box activators (CLOCK/NPAS2 and BMAL1) and RRE repressors (REV-ERBs), but not the D-box repressor (E4BP4) or RRE activator (ROR *α*), bind the *Dgat2* promoter. The choice of *Dgat2* is also supported by an independent RNAi study [53] performed by Maier and colleagues (personal communication), where a significant one hour period lengthening was observed in U2OS cells in response to the gene knockdown. This RNAi phenotype led to the choice of *Dgat2* although it was not robust to the choice of weighting scheme (Table S1 in Additional file 2).

Next, we consider a protein phosphatase (PP) regulatory subunit 3B of PP1 *Ppp1r3b* that was a robust candidate gene. PP1R3B suppresses the glycogen phosphorylase activity of PP1 and enhanced its glycogen synthase activity. Although *Ppp1r3b* was expressed in a circadian manner in fewer tissues than the previous four genes, the knockdown of the gene displayed a significant long period phenotype (2.5 h lengthening) in an independent RNAi study [53] on human U2OS cells. PP1, a known clock component [37], affects the period of the mammalian circadian clock [54, 55] and is known to target PER1 [56]. Nevertheless, none of the catalytic subunits of PP1 is transcriptionally circadian in any tissue. This might indeed be novel layer of the clock regulation by PP1 by means of a circadian regulatory subunit *Ppp1r3b*.

Finally, Uridine phosphorylase 2 (*Upp2*) was a robust candidate gene in our meta-analysis. *Upp2* is a particularly interesting candidate, since it is an example of a circadian protein that is also a metabolic enzyme. Eckel-Mahan et al. [40] showed that *Upp2* acted on two circadian metabolites (Uridine and Uracil) and thus is one of the few known common nodes between circadian transcriptome and circadian metabolome [41, 57]. While *Upp2* has been shown to be a clock output gene in the liver [40], our meta-analysis revealed that *Upp2* was robustly circadian in the kidney too with the same phase as in the liver (∼CT10). Interestingly, in humans, *Upp2* is expressed more in the kidney than in the liver [58]. Therefore, it might be interesting to study the circadian role of *Upp2* further, in addition to the known metabolic context in the liver.

## Conclusion

We combined multiple high-throughput public data sources of circadian data to filter the shortlist of 1000 potential circadian genes from [9] to obtain 11 novel robust candidate genes. In particular, we suggested *Por*, *Mtss1*, *Pim3*, *Dgat2*, *Ppp1r3b* and *Upp2* for further experimental studies as potential clock genes. These genes appear to have a role in either metabolism or cancer or stress response making them a potential link between the circadian clock and these physiological processes. We also showed from the different ChIP-seq data that phase regulation of transcription of circadian genes is driven by specific transcription factors in a combinatorial manner consistent across multiple tissues.

Although we focused our discussion on the significant candidate genes, we have assigned scores based on our analysis for the all 1000 shortlisted genes that can serve as a starting point to integrate additional data sources as they or tools to integrate them (for e.g., to integrate metabolomics data [40, 41, 57]) become available. In order to keep the scoring in the meta-analysis simple we used binary scoring of hits from various data sources. A Bayesian analysis might be performed in order to consistently combine final scores from [9] for a better consolidated final ranking. Meta-analyses of the ever growing publicly available data targeted at different aspects of the regulatory network has the potential to aid functional network discovery in the circadian and other contexts and utilize these data to their fullest potential.

## Availability of data and materials

The data sets supporting the results of this article are listed in Additional file 4.

## Additional files

Additional file 1
**Complete score table from the meta-analysis for all the 1000 master list genes with individual hits of a gene within each data set.** (XLSX 98 kb)

Additional file 2
**Supplementary Tables.** **Table S1.** List of core circadian genes according to Anafi et al. [9], Wallach et al. [28] and Ukai and Ueda [37] in the master list of 1000 genes and their scores based on our meta-analysis. The shaded genes were found among the 31 candidate genes by our meta-analysis. **Table S2.** List of candidate genes robust to choice of weighting scheme. **Table S3.** Highly significant candidate clock associated genes with the number tissues in which they are known to be expressed in a circadian manner. **Table S4.** Peak protein expression phase of different circadian transcription factors. (PDF 115 kb)

Additional file 3
**Supplementary Figures.** **Figure S1.** The distribution of promoter elements among the master list genes. **Figure S2.** Distribution of total scores for each gene in the 1000 gene-long master list. (PDF 31 kb)

Additional file 4
**Sources of publicly-available data used in the meta-analysis.** (PDF 48 kb)

## Abbreviations

ChIP-seq: Chromatin immunoprecipitation sequencing
CT: Circadian time
E-box: Enhancer box
FDR: False discovery rate
GLM: Generalized linear model
PPI: Protein-protein interaction
RNAi: Ribonucleic acid interference
RRE: ROR response element
SCN: Suprachiasmatic nucleus
SILAC: Stable isotope labeling by amino acids in cell culture
siRNA: small interference RNA
TF: Transcription factor
TTFL: Transcriptional-translational feedback loop
ZT: Zeitgeber time
