# Supplementary material for: Mining for novel candidate clock genes in the circadian regulatory network
Source: BMC Syst Biol. 2015 Nov 14;9:78. doi: 10.1186/s12918-015-0227-2 (PMC4650315; doi:10.1186/s12918-015-0227-2)
Supplement: Additional file 2 — Supplementary Tables. Table S1. List of core circadian genes according to Anafi et al. [9], Wallach et al. [28] and Ukai and Ueda [37] in the master list of 1000 genes and their scores based on our meta-analysis. The shaded genes were found among the 31 candidate genes by our meta-analysis. Table S2. List of candidate genes robust to choice of weighting scheme. Table S3. Highly significant candidate clock associated genes with the number tissues in which they are known to be expressed in a circadian manner. Table S4. Peak protein expression phase of different circadian transcription factors. (PDF 115 kb) [file 12918_2015_227_MOESM2_ESM.pdf]

Supplementary Tables

Table S1: List of 31 candidate genes with significant total scores ($p < 0.001$), i.e., total score of 13 or more, produced by our meta-analysis. This list includes both genes that are and are *not* robust to the choice of weighting schemes. The non-robust genes are shown in bold.

Gene	Score
Per1	16
Por	16
Gne	16
Nfil3	15
Etnk2	15
Tef	15
Azin1	15
Lima1	15
Upp2	14
Cry2	14
Dbp	14
Fam20a	14
Mtss1	14
Hlf	14
Nr1d1	14
Gys2	14
Usp2	14
Tomm20	14
Bhlhe40	13
Ppp1r3b	13
Chka	13
Mafb	13
Pik3r1	13
Ppm1a	13
Fam160b1	13
Pdk1	13
Eva1a	13
Phf17	13
Arhgef26	13
Dgat2	13
Pim3	13

Table S2: List of core circadian genes according to Anafi et al., Wallach et al. and Ukai and Ueda in the master list of 1000 genes and their scores based on our meta-analysis. The shaded genes were found among the 31 candidate genes by our meta-analysis.

Gene	Score	Rank (Anafi et al.)	Anafi exemplar gene	Wallach clock gene	Ueda clock gene
Per1	16	224	✓	✓	✓
Nfil3	15	11	✗	✓	✓
Tef	15	18	✗	✗	✓
Hlf	14	354	✗	✗	✓
Cry2	14	162	✓	✓	✓
Nr1d1	14	4	✓	✓	✓
Dbp	14	37	✗	✓	✓
Bhlhe40	13	777	✗	✓	✓
Per2	12	7	✓	✓	✓
Cry1	11	2	✓	✓	✓
Nr1d2	11	5	✓	✓	✓
Npas2	9	10	✓	✓	✓
Rorc	9	6	✓	✓	✓
Clock	8	8	✓	✓	✓
Per3	8	3	✓	✓	✓
Arntl	6	1	✓	✓	✓
Ppp2r1b	4	783	✗	✓	✗
Rora	3	19	✓	✓	✓
Nono	2	711	✗	✓	✗
Csnk2a1	2	477	✗	✓	✗
Rorb	1	961	✓	✓	✓

Table S3: Highly significant candidate clock associated genes (both robust and non-robust to weighting scheme) and number of tissues in which they are expressed in a circadian manner (JTK$_{qvalue}$ < 0.05). When genes are expressed in multiple tissues in [1], their mean phase of expression, concentration measure R [2] and p-value for the coherence of phase of expression based on the Rayleigh-test are also shown. Already known clock genes are highlighted in grey.

Symbol	Score	No. of tissues	Mean phase (CT)	Std. dev. phase (CT)	p-value	Robust
Dbp	14	14	10.32	0.97	9.7×10^{-7}	✓
Nr1d1	14	14	7.46	0.93	1×10^{-6}	✓
Hlf	14	12	12.14	0.90	$< 1 \times 10^{-8}$	✓
Tef	15	12	11.75	0.98	1.4×10^{-6}	✓
Nfil3	15	10	21.23	0.93	$< 1 \times 10^{-8}$	✓
Usp2	14	10	12.15	0.98	$< 1 \times 10^{-8}$	✓
Por	16	9	10.75	0.94	$< 1 \times 10^{-8}$	✓
Cry2	14	8	11.69	0.88	3.7×10^{-4}	✓
Per1	16	8	10.37	0.94	5.8×10^{-6}	✓
Dgat2	13	6	11.10	0.78	1.7×10^{-2}	✗
Pim3	13	6	9.31	0.94	9.3×10^{-4}	✗
Phf17	13	5	6.81	0.78	3.9×10^{-2}	✗
Pik3r1	13	5	17.30	0.74	5.8×10^{-2}	✗
Bhlhe40	13	4	11.31	0.90	2.6×10^{-2}	✓
Chka	13	4	18.40	0.67	0.18	✗
Mtss1	14	4	8.56	0.44	0.49	✓
Fam20a	14	3	1.56	0.92	6.6×10^{-2}	✓
Azin1	15	2	6.26	0.07	0.99	✓
Ppp1r3b	13	2	17.31	0.98	0.15	✓
Upp2	15	2	10.00	0.97	0.16	✓
Etnk2	15	1	12.00			✓
Fam176a	13	1	17.54			✗
Gne	16	1	10.50			✓
Gys2	14	1	14.00			✓
Lima1	15	1	11.00			✓
Mafb	13	1	10.50			✗
Ppm1a	13	1	9.00			✗

Table S4: Peak protein expression phase of different circadian transcription factors.

Circadian transcription factor	Type	Peak protein phase (CT)	Reference
CLOCK	activator	6	[3]
BMAL1	activator	7	[3]
NPAS2	activator	8	[3]
REV-ERBα, β	repressor	10	[4]
RORα	activator	20-22	[5]
E4BP4	repressor	20-22	[5]

References

[1] Zhang, R., Lahens, N.F., Ballance, H.I., Hughes, M.E., Hogenesch, J.B.: A circadian gene expression atlas in mammals: Implications for biology and medicine. Proceedings of the National Academy of Sciences **111**(45), 16219–16224 (2014). doi:10.1073/pnas.1408886111

[2] Jammalamadaka, S.R., Sengupta, A.: Topics in Circular Statistics vol. 5. World Scientific Publishing, Singapore (2001)

[3] Koike, N., Yoo, S.-H., Huang, H.-C., Kumar, V., Lee, C., Kim, T.-K., Takahashi, J.S.: Transcriptional Architecture and Chromatin Landscape of the Core Circadian Clock in Mammals. Science **338**(6105), 349–354 (2012). doi:10.1126/science.1226339

[4] Bugge, A., Feng, D., Everett, L.J., Briggs, E.R., Mullican, S.E., Wang, F., Jager, J., Lazar, M.A.: Rev-erbα and Rev-erbβ coordinately protect the circadian clock and normal metabolic function. Genes Dev. **26**(7), 657–667 (2012). doi:10.1101/gad.186858.112

[5] Fang, B., Everett, L.J., Jager, J., Briggs, E., Armour, S.M., Feng, D., Roy, A., Gerhart-Hines, Z., Sun, Z., Lazar, M.A.: Circadian Enhancers Coordinate Multiple Phases of Rhythmic Gene Transcription In Vivo. Cell **159**(5), 1140–1152 (2014). doi:10.1016/j.cell.2014.10.022
