# Supplementary material for: Mining for novel candidate clock genes in the circadian regulatory network
Source: BMC Syst Biol. 2015 Nov 14;9:78. doi: 10.1186/s12918-015-0227-2 (PMC4650315; doi:10.1186/s12918-015-0227-2)
Supplement: Additional file 3 — Supplementary Figures. Figure S1. The distribution of promoter elements among the master list genes. Figure S2. Distribution of total scores for each gene in the 1000 gene-long master list. (PDF 31 kb) [file 12918_2015_227_MOESM3_ESM.pdf]

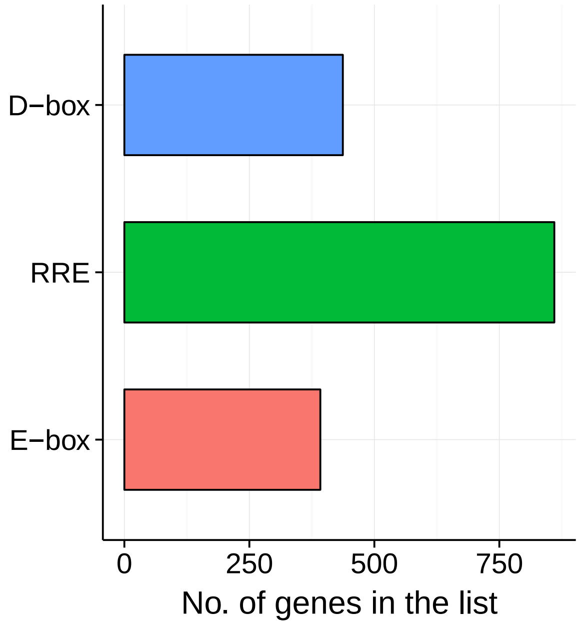

Figure S1: The distribution of promoter elements among the master list genes based on the ChIP-seq data of TFs (E-box: CLOCK, BMAL1, NPAS2; D-box: E4BP4; RRE: REV-ERBα, β, RORα).

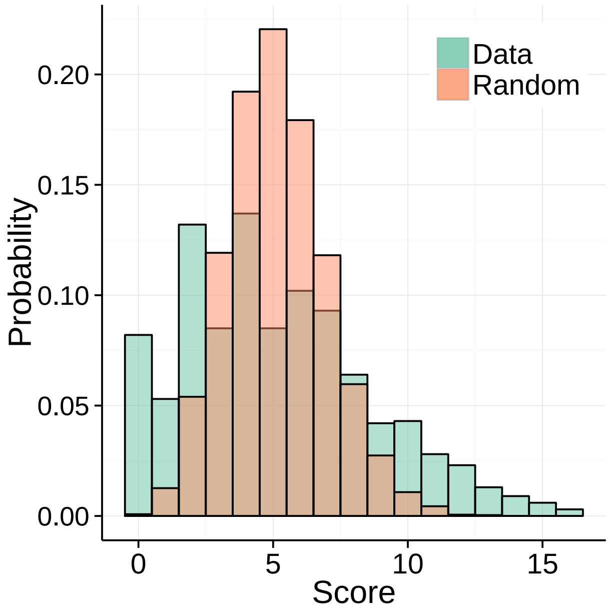

Figure S2: The distribution of total scores for each gene in the 1000 gene-long master list (in green) compared against the empirical distribution of total scores based on the randomized shuffling procedure described in the Method section.
