# Supplementary material for: Mining for novel candidate clock genes in the circadian regulatory network
Source: BMC Syst Biol. 2015 Nov 14;9:78. doi: 10.1186/s12918-015-0227-2 (PMC4650315; doi:10.1186/s12918-015-0227-2)
Supplement: Additional file 4 — Sources of publicly-available data used in the meta-analysis. (PDF 48 kb) [file 12918_2015_227_MOESM4_ESM.pdf]

Publically-available data sources used in this study with hyperlinks (all links accessed on 2.11.2015).

Type	Source	Location	Hyperlink/DOI/Accession Number
Master list of genes	Anafi et al. [1]	Table S2	doi:10.1371/journal.pbio.1001840.s010
	Koike et al. [2]	Table S2	http://www.sciencemag.org/content/suppl/2012/08/29/science.1226339.DC1/Koike_TableS2_MasterPeakList_072612.xlsx
	Rey et al. [3]	Text S2	doi: 10.1371/journal.pbio.1000595.s019
ChIP-seq	Cho et al. [4]	GEO [5]	Accession no: GSE34019
	Bugge et al. [6]	GEO [5]	Accession no: GSE36375
	Feng et al. [7]		Accession no: GSE26345
	Fang et al. [8]	GEO [5]	Accession no: GSE59486
	Robles et al. [9]	Table S2	doi:10.1371/journal.pgen.1004047.s007
Proteomics	Mauvoisin et al. [10]	Dataset S1	http://www.pnas.org/content/suppl/2013/12/16/1314066111.DCSupplemental/sd01.xls
	Chiang et al. [11]	Table S2	doi:10.1371/journal.pgen.1004695.s006
Protein-protein interaction	Wallach et al. [12]	Table S1	doi:10.1371/journal.pgen.1003398.s008
	PINA mouse database [13]		http://cbg.garvan.unsw.edu.au/pina/download/Mus%20musculus-20140521.tsv
Multi-tissue mRNA transcript profiles	Zhang et al.[14]	GEO [5]	Accession no: GSE54650

References

[1] Anafi, R.C., Lee, Y., Sato, T.K., Venkataraman, A., Ramanathan, C., Kavakli, I.H., Hughes, M.E., Baggs, J.E., Growe, J., Liu, A.C., Kim, J., Hogenesch, J.B.: Machine Learning Helps Identify CHRONO as a Circadian Clock Component. PLoS Biol **12**(4), 1001840 (2014). doi:10.1371/journal.pbio.1001840

[2] Koike, N., Yoo, S.-H., Huang, H.-C., Kumar, V., Lee, C., Kim, T.-K., Takahashi, J.S.: Transcriptional Architecture and Chromatin Landscape of the Core Circadian Clock in Mammals. Science **338**(6105), 349–354 (2012). doi:10.1126/science.1226339

[3] Rey, G., Cesbron, F., Rougemont, J., Reinke, H., Brunner, M., Naef, F.: Genome-Wide and Phase-Specific DNA-Binding Rhythms of BMAL1 Control Circadian Output Functions in Mouse Liver. PLoS Biol **9**(2), 1000595 (2011). doi:10.1371/journal.pbio.1000595

[4] Cho, H., Zhao, X., Hatori, M., Yu, R.T., Barish, G.D., Lam, M.T., Chong, L.-W., DiTacchio, L., Atkins, A.R., Glass, C.K., Liddle, C., Auwerx, J., Downes, M., Panda, S., Evans, R.M.: Regulation of circadian behaviour and metabolism by REV-ERB-α and REV-ERB-β. Nature **485**(7396), 123–127 (2012). doi:10.1038/nature11048

[5] The Gene Expression Omnibus (GEO). http://www.ncbi.nlm.nih.gov/geo/ Accessed 2015-10-31

[6] Bugge, A., Feng, D., Everett, L.J., Briggs, E.R., Mullican, S.E., Wang, F., Jager, J., Lazar, M.A.: Rev-erbα and Rev-erbβ coordinately protect the circadian clock and normal metabolic function. Genes Dev. **26**(7), 657–667 (2012). doi:10.1101/gad.186858.112

[7] Feng, D., Liu, T., Sun, Z., Bugge, A., Mullican, S.E., Alenghat, T., Liu, X.S., Lazar, M.A.: A circadian rhythm orchestrated by histone deacetylase 3 controls hepatic lipid metabolism. Science **331**(6022), 1315–1319 (2011). doi:10.1126/science.1198125

[8] Fang, B., Everett, L.J., Jager, J., Briggs, E., Armour, S.M., Feng, D., Roy, A., Gerhart-Hines, Z., Sun, Z., Lazar, M.A.: Circadian Enhancers Coordinate Multiple Phases of Rhythmic Gene Transcription In Vivo. Cell **159**(5), 1140–1152 (2014). doi:10.1016/j.cell.2014.10.022

[9] Robles, M.S., Cox, J., Mann, M.: In-Vivo Quantitative Proteomics Reveals a Key Contribution of Post-Transcriptional Mechanisms to the Circadian Regulation of Liver Metabolism. PLoS Genet **10**(1), 1004047 (2014). doi:10.1371/journal.pgen.1004047

[10] Mauvoisin, D., Wang, J., Jouffe, C., Martin, E., Atger, F., Waridel, P., Quadroni, M., Gachon, F., Naef, F.: Circadian clock-dependent and -independent rhythmic proteomes implement distinct diurnal functions in mouse liver. PNAS **111**(1), 167–172 (2014). doi:10.1073/pnas.1314066111

[11] Chiang, C.-K., Mehta, N., Patel, A., Zhang, P., Ning, Z., Mayne, J., Sun, W.Y.L., Cheng, H.-Y.M., Figeys, D.: The Proteomic Landscape of the Suprachiasmatic Nucleus Clock Reveals Large-Scale Coordination of Key Biological Processes. PLoS Genet **10**(10), 1004695 (2014). doi:10.1371/journal.pgen.1004695

[12] Wallach, T., Schellenberg, K., Maier, B., Kalathur, R.K.R., Porras, P., Wanker, E.E., Futschik, M.E., Kramer, A.: Dynamic Circadian Protein–Protein Interaction Networks Predict Temporal Organization of Cellular Functions. PLoS Genet **9**(3), 1003398 (2013). doi:10.1371/journal.pgen.1003398

[13] Protein Interaction Network Analysis Platform (PINA). http://cbg.garvan.unsw.edu.au/pina/ Accessed 2015-3-31

[14] Zhang, R., Lahens, N.F., Ballance, H.I., Hughes, M.E., Hogenesch, J.B.: A circadian gene expression atlas in mammals: Implications for biology and medicine. Proceedings of the National Academy of Sciences **111**(45), 16219–16224 (2014). doi:10.1073/pnas.1408886111
